# Laws of New York of Interest to Physicians

**Published:** 1914-09

**Authors:** 


### Laws of New York of Interest to Physicians.

Chap. 365, Section 1. Chapter forty-nine of the laws of nineteen hundred and nine, entitled "An act in relation to the public health, constituting chapter forty-five of the consolidated laws," is hereby amended by inserting therein a new article, to be article seventeen-a, to read as follows: Article 17-a. Suppression of Certain Nuisances.

§ 343-a. **Nuisance defined.** For the purpose of this article any house, building, place or any separate part or portion thereof, or the ground itself, in or upon which assignation or prostitution is conducted, practiced, permitted, carried on or exists is declared a nuisance, and whoever knowingly shall erect, establish, permit, continue, maintain, own, lease or sublease any house, building, erection, place or any separate part or portion thereof, used for such purpose, shall be guilty of maintaining a nuisance.

(The remaining sections regarding methods of procedure, etc., are omitted here).

Chap. 323, § 45. **Establishment of county hospital for tuberculosis.** The board of supervisors of any county shall have power by a majority vote to establish a county hospital for the care and treatment of persons suffering from the disease known as tuberculosis. The board of supervisors of any county that previous to January first, nineteen hundred and fourteen, has not voted to establish a hospital shall have authority to submit the question of establishing such a hospital to the voters of the county at any general election at which public officers are elected. The board of supervisors shall fix the sum of money deemed necessary for the establishment of said hospital. The form of the proposition submitted shall read as follows: "Shall the county of ......appropriate the sum of ...... dollars for the establishment of a tuberculosis hospital." The notices of the general election shall state that the proposition will be voted upon and in the form set forth above. Provision for taking such vote and for the canvassing and returning of the result shall be made by the duly constituted election authorities.

If a majority of the voters voting on such proposition shall vote in favor thereof then such hospital shall be established hereunder and the sum of money named in the said proposition shall be deemed appropriated, and it shall be the duty of the board of supervisors to proceed forthwith to exercise the powers and authority conferred upon it in this section.

When the board of supervisors of any county shall have voted to establish such hospital, or when a referendum on the proposition of establishing such a hospital in a county, as authorized above, shall have been carried, the board of supervisors shall:

1. Purchase or lease real property therefor, or acquire such real property, and easements therein, by condemnation proceedings, in the manner prescribed by the condemnation law, in any town, city or village in the county. After the presentation of the petition in such proceeding prescribed in section three thousand three hundred and sixty of the code of civil procedure and the filing of the notice of pendency of action prescribed in section three thousand three hundred and eighty-one thereof, said board of supervisors shall be and become seized of the whole or such part of the real property described in said petition to be so acquired for carrying into effect the provisions of this act, as such board may, by resolution adopted at a regular or special session, determine to be necessary for the immediate use, and such board for and in the name of such county may enter upon, occupy and use such real property so described and required for such purposes. Such resolution shall contain a description of the real property of which possession is to be taken and the day upon which possession will be taken. Said board of supervisors shall cause a copy of such resolution to be filed in the county clerk's office of the county in which such property is situate, and notice of the adoption thereof, with a copy of the resolution and of its intention to take possession of the premises therein described on a day certain, also therein named, to be served, either personally or by mail, upon the owner or owners of, and persons interested in such real property, at least five days prior to the day fixed in such resolution for taking possession. From the time of the service of such notice, the entry upon and appropriation by the county of the real property therein described for the purposes provided for by this act, shall be deemed complete, and such notice so served shall be conclusive evidence of such entry and appropriation and of the quantity and boundaries of the lands appropriated. The board of supervisors may cause a duplicate copy of such papers so served, with an affidavit of due service thereof on such owner or person interested, to be recorded in the books used for recording deeds in the office of the county clerk of its county, and the record of such notice and such proof of service shall be prima facie evidence of the due service thereof. Compensation for property thus acquired shall be made in such condemnation proceeding.

2. Erect all necessary buildings and alter any buildings, on the property when acquired for the use of said hospital, provided that the plans for such erection or alteration shall first be approved by the state commissioner of health.

3. Cause to be assessed, levied and collected such sums of money as it shall deem necessary for suitable lands, buildings and improvements for said hospital, and for the maintenance thereof, and for all other necessary expenditures therefor; and to borrow money for the erection of such hospital and for the purchase of a site therefor on the credit of the county, and issue county obligations therefor, in such manner as it may do for other county purposes.

4. Appoint a board of managers for said hospital as hereinafter provided.

5. Accept and hold in trust for the county, any grant or devise of land, or any gift or bequest of money or other personal property, or any donation to be applied, principal or income, or both, for the benefit of said hospital, and apply the same in accordance with the terms of the gift.

§ 2. Section forty-seven of such chapter as added by chapter three hundred and forty-one of the laws of nineteen hundred and nine, and amended by chapters forty and three hundred and seventy-nine of the laws of nineteen hundred and thirteen, is hereby amended by adding thereto a new subdivision to be subdivision nine, to read as follows:

9. Shall have authority to employ a county nurse or nurses for discovery of tuberculosis cases and for the visitation of such cases and of patients discharged from the hospital and for such other duties as may seem appropriate; and may cause to be examined by the superintendent or one of his medical staff suspected cases of tuberculosis reported to it by the county nurse or nurses or by physicians, teachers, employers, heads of families, or others; and to take such other steps for the care, treatment, and prevention of tuberculosis as it may from time to time deem wise.

§ 3. This act shall take effect immediately.

Chap. 319, An Act to amend the public health law, relative to qualifications to practice medicine. Section 1. Section 161 of chapter 49 of the laws of 1909, entitled "An act in relation to the public health, constituting chapter 45 of the consolidated laws," is hereby amended to read as follows:

§ 161. **Qualifications.** No person shall practice medicine, unless registered and legally authorized prior to September first, eighteen hundred and ninety-one, or unless licensed by the regents and registered under article eight of chapter six hundred and sixty-one of the laws of eighteen hundred and ninety-three and acts amendatory thereto, or unless licensed by the regents and registered as required by this article; nor shall any person practice under this article who has ever been convicted of a felony by any court, or whose authority to practice is suspended or revoked by the regents on recommendation of the state board. The conviction of a felony shall include the conviction of any offense which if committed within the state of New York would constitute a felony under the laws thereof. If a person convicted of a felony is subsequently pardoned by the governor of the state where such conviction was had, or by the president of the United States, the regents may, in their discretion, on application of such person, and on the submission to them of satisfactory evidence, restore to such person the right to practice medicine in this state, unless such conviction has been for misconduct in his professional capacity.

§2. This act shall take effect immediately.

Chap. 363, An Act to amend the public health law, in relation to the sale of habit-forming drugs. Article 11-a. Habit Forming Drugs.

§ 245. **Sale prohibited; exception.** No pharmacist, druggist or other person shall sell, have or offer for sale or give away any chloral, opium or any of its salts, alkaloids or derivatives or any compound of preparation of any of them except upon the written prescription of a duly licensed physician, veterinarian or dentist, provided that the provisions of this article shall not apply to the sale of domestic and proprietary remedies, actually sold in good faith as medicines and not for the purpose of evading the provisions of this article and provided further that such remedies and preparations do not contain more than two grains of opium, or one-fourth grain of morphine or one-fourth grain of heroin or one grain of codeine, or ten grains of chloral or their salts in one fluid ounce or if a solid preparation, in one avoirdupois ounce, nor to plasters, liniments and ointments for external use only.

§ 246. **Prescriptions; certificates.** It shall be unlawful for any person to sell at retail or give away any of the drugs, their salts, derivatives or preparations mentioned in section two hundred and forty-five of this chapter except as herein provided without first receiving a written prescription signed by a duly licensed physician, veterinarian or dentist. The prescription must contain substantially the following: The name in full of the physician, veterinarian or dentist issuing such prescription, his office address, his office hours, and telephone, and the name, age and address of the person to whom and date on which such prescription is issued. It shall be unlawful for any duly licensed physician, veterinarian or dentist to issue any such prescription containing any of the drugs, their salts, derivatives or preparations mentioned in section two hundred and forty-five of this chapter except after a physical examination of any person for the treatment of disease, injury or deformity. It shall be unlawful for any person to sell at retail any of the drugs or preparations of any of those mentioned in section two hundred and forty-five of this article without first verifying the authority of any prescription containing more than four grains of morphine, thirty grains of opium, two grains of heroin, six grains of codeine or four drams of chloral. Such verification can be made by telephone or otherwise. Such prescription so received shall be filled out at the time of receiving the same for the full quantity prescribed and no prescription so received shall be filled out more than ten days after the date which said prescription be dated. Such prescription, from which no copy shall be taken, shall be retained by the person who dispenses the same and shall be filled but once. Such prescription shall be kept on the general prescription file and given a regular consecutive number on such file. On such prescription shall be inscribed the name and address of the purchaser making such purchase and the date upon which said sale is made. Any person who sells at retail, furnishes or dispenses any of the drugs mentioned in section two hundred and forty-five of this chapter upon a written prescription by a duly registered physician or veterinarian or dentist shall at the time of dispensing the same, place upon the package a label or deliver therewith a certificate stating the name and address of the person selling or furnishing the same, the name and address of the physician, veterinarian or dentist upon whose prescription such sale is made, the date of sale, and the name of the person to whom such sale is made. Any person, other than a manufacturer of any of the drugs mentioned in section two hundred and forty-five or a wholesale dealer in drugs or a licensed pharmacist, licensed druggist, duly registered practicing physician, licensed veterinarian or a licensed dentist, who shall possess any of the drugs mentioned in section two hundred and forty-five or their salts, derivatives or prescriptions, shall be guilty of a misdemeanor, unless said possession is authorized by the certificate described in this section. Nothing herein contained shall be construed to prohibit the sale of any of such drugs by any manufacturing pharmacists or chemists or wholesale or retail pharmacists or druggists, or to hospitals, colleges, scientific or public institutions, except that such sales shall be made in the manner provided in the next succeeding section.

§ 247. **Order blanks; filing.** The state commissioner of health shall prepare and furnish to all boards of health or officers official order blanks, serially numbered in duplicate, bound in book form, with carbon or transfer paper between the duplicate pages. The said official order shall be furnished by the local health board or officer to any local, duly licensed physician, dentist, pharmacist, druggist or veterinarian, upon which must be written all orders for the purchase of any of the drugs enumerated in section two hundred and forty-five of this chapter for the use of such physician, dentist, pharmacist, druggist or veterinarian. It shall be unlawful for any person to sell, furnish or dispose to any physician, pharmacist, druggist, veterinarian or dentist any of the drugs enumerated in section two hundred and forty-five of this chapter without first receiving from such physician, druggist, veterinarian or dentist an official order blank as provided in this section, which official order shall be retained by the person or corporation who sells, furnishes or dispenses any of the drugs enumerated in section two hundred and forty-five of this chapter, and such official order shall be kept in a separate file or book and an entry made or caused to be made on the order stating the date of sale, the name and address of the purchaser and the name of the person making such sale.

§ 248. **Physicians, et cetera, to keep records.** All physicians, druggists, pharmacists, veterinarians and dentists shall keep on record the name and address of each person to whom such physician, dentist or veterinarian administers or disposes in any way whatsoever any of the drugs enumerated in section two hundred and forty-five of this chapter, and the quantity so administered, disposed of or given away. Such record shall be preserved for five years and shall always be open for inspection by the proper authorities. Any violation of this section is hereby declared to be a misdemeanor.

§ 249. **Hypodermic syringe; sale of; record; penalty.** It is unlawful for any person to sell at retail or to furnish to any person other than a duly licensed physician, dentist, or veterinarian, an instrument commonly known as a hypodermic syringe or an instrument commonly known as a hypodermic needle, without the written order of a duly licensed physician or veterinarian. Every person who disposes of or sells at retail, or furnishes or gives away to any person, either of the above instruments, upon the written order of a duly licensed physician or veterinarian, shall, before delivering the same, enter in a book kept for that purpose the date of the sale, the name and address of the purchaser, and a description of the instrument sold, disposed of, furnished or given away. Any person or persons who sell, dispose of or give away an instrument commonly known as a hypodermic syringe, or an instrument commonly known as a hypodermic needle, except in the manner prescribed in this section, shall be guilty of a misdemeanor.

§ 249-a. **Commitment of habitual drug users; procedure; discharge.** The constant use by any person of any habit-forming drug, except under the direction and consent of a duly licensed physician, is hereby declared to be dangerous to the public health. Whenever a complaint shall be made to any magistrate that any person is addicted to the use of any habitforming drug, without the consent or direction of a duly licensed physician, such magistrate, after due notice and hearing, if satisfied that the complaint is founded and that the person is addicted to the use of a habit-forming drug, shall commit such person to a state, county or city hospital or institutions licensed under the state lunacy commission. Whenever the chief medical officer of such institution shall certify to any magistrate that any person so committed has been sufficiently treated or give any other reason which is deemed adequate and sufficient, he may discharge the person so committed. Every person committed under the provisions of this section shall observe all the rules and regulations of the institution or hospital. Any such person who wilfully violates the rules and regulations of the institution or repeatedly conducts himself in a disorderly manner may be taken before a magistrate by the order of the chief medical officer of the institution. The chief medical officer may enter a complaint against such person for disorderly conduct and the magistrate, after a hearing and upon due evidence of such disorderly conduct, may commit such person for a period of not to exceed six months to any institution to which persons convicted of disorderly conduct or vagrancy may be committed, and such institution shall keep such persons separate and apart from the other inmates, provided that nothing in this section shall be construed to prohibit any person committed to any institution under its provisions from appealing to any court having jurisdiction for a review of the evidence in which this commitment was made.

§ 249-b. **Revocation of licenses.** Any license heretofore issued to any physician, dentist, veterinarian, pharmacist or registered nurse may be revoked by the proper officers or boards having power to issue licenses to any of the foregoing upon proof that the licensee is addicted to the use of any habit-forming drug or drugs after giving such licensee reasonable notice and opportunity to be heard. Whenever it shall appear after one year from date of revocation of such license that such licensee has fully recovered and is no longer an addict to any of the drugs herein prohibited, such board may grant a rehearing and in its discretion reissue the license of such licensee.

§ 249-c. **Revocation of license after conviction.** Whenever any physician, dentist, veterinarian, pharmacist or registered nurse is convicted in a court having jurisdiction of any of the violations of this article, any officer or board having power to issue licenses to any such physician, dentist, veterinarian, pharmacist, or registered nurse may, after giving such licensee reasonable notice and opportunity to be heard, revoke the same.

§ 249-d. **Penalties.** Any violation of any of the provisions of this article shall be deemed a misdemeanor. Nothing contained in this article shall be construed to amend or repeal section seventeen hundred and forty-six of the penal law.

§ 2. This act shall take effect July 1, 1914.

Chap. 414, An Act to amend the public health law, in relation to cold storage.

§ 336. **Cold storage food to be marked.** It shall hereafter be unlawful for any person or persons, corporation or corporations, engaged in the business of cold storage warehousemen or in the business of refrigerating, to receive any kind of food unless the said food is in an apparently pure and wholesome condition, and the food or the package containing the same is branded, stamped or marked, in some conspicuous place, with the day, month and year, when the same is received in storage or refrigeration.

It shall be unlawful for any person or persons, corporation or corporations, engaged in the business of cold storage warehousemen or in the business of refrigerating to permit any article of any kind whatsoever used for food in the possession of any person or persons, corporation or corporations, engaged in the business of cold storage warehousemen or refrigerating, to be taken from their possession without first having branded, stamped or marked on said food stuffs or the package containing same, in a conspicuous place, the day, month and year, when said food stuffs or package was removed from cold storage or refrigeration.

It shall also be unlawful for any person or persons, corporation or corporations, to offer for storage in a cold storage warehouse or to place in storage in a cold storage warehouse any article of food unless the same is in an apparently pure and wholesome condition.

§ 337. **Time that cold storage foods may be kept.** It shall hereafter be unlawful for any person, corporation or corporations, engaged in the business of cold storage warehousemen or refrigerating, or for any person placing food in a cold storage warehouse, to keep in storage for preservation or otherwise any kind of food or any article used for food a longer period than ten calendar months, excepting butter products which may be kept in said cold storage or refrigeration twelve calendar months.

§ 338. **Powers of state commissioner of health.** The state commissioner of health is hereby vested with full power and authority to inspect and supervise all places in this state now used or hereafter to be used for cold storage or refrigerating purposes; the state commissioner of health or his duly authorized agents or employees shall be permitted access to such place or places and all parts thereof at all times for the purpose of seeing that said place or places are kept and maintained in a clean and sanitary manner, and for the purpose of determining whether or not the provisions of this article or any other act relating to food stuffs are being complied with. The commissioner of health shall have the power by subpoena or subpoena duces tecum, issued and attested by him in his official capacity to require the attendance and testimony before him, or the deputy commissioner, of any person who he may have reason to believe has knowledge of any alleged violation of this article, and the production before him, or the deputy commissioner, of any records, books, papers and documents for the purpose of investigating any alleged violation of this article. Such subpoenas or subpoenas duces tecum may be served by any person over the age of twenty-one years. No person shall be excused from attending and testifying or producing any records, books, papers or other documents before said commissioner of health, or the deputy commissioner, upon such investigation upon the ground or for the reason that the testimony or evidence, documentary or otherwise, required of him may tend to convict him of a crime or subject him to a penalty or forfeiture, but no person shall be prosecuted or subjected to any penalty or forfeiture for or on account of any transaction, matter or thing concerning which he may so testify or produce evidence, documentary or otherwise, and no testimony so given or produced shall be received against him upon any criminal action, investigation or proceeding. Any person who shall omit, neglect or refuse to attend and testify or to produce any records, books, papers or documents, if in his power so to do, in obedience to such subpoena or subpoena duces tecum shall be guilty of a misdemeanor. Any person who shall willfully and knowingly make any false statement under oath before the commissioner of health, or the deputy commissioner, concerning a material matter shall be guilty of perjury. The commissioner of health and the deputy commissioner are hereby authorized and empowered to administer oaths and affirmations in the usual appropriate forms to any person in any matter or proceedings authorized as aforesaid and in all matters pertaining or relating to this article and to take and administer oaths and affirmations in the usual appropriate forms, in taking any affidavit or deposition which may be necessary or required by law or by any order, rule or regulation of the commissioner of health for or in connection with the official purposes, affairs, powers, duties or proceedings of said commissioner of health, or the deputy commissioner, or for any official purpose lawfully authorized by said commissioner of health. The power of supervision hereby granted shall extend to enable the state commissioner of health to adopt such reasonable rules and regulations as may be determined upon from time to time as essential to the proper protection of the consumer of the commodities kept and preserved in such place or places, and the state commissioner of health may appoint and designate from time to time such person or persons as he deems fit for the purpose of making such inspections.

§ 2. This act shall take effect immediately.

Chap. 317, An Act to amend the public health law, relative to the registration of licenses to practice chiropody.

§ 278. **Practicing without registering prohibited.** Every license to practice chiropody before the licensee begins practicing thereunder shall be registered in a book kept in the clerk's office of the county where such practice is to be carried on, with the name, the residence, the place and date of birth, and the source, the number and date of his license to practice. Before registering, each licensee shall file, to be kept in a bound volume in a county clerk's office, an affidavit of the above facts, and also that he is the person named in such license and had before receiving the same complied with all requirements as to attendance and amount of study and examinations required by law and the rules of the university as preliminary to the conferment thereof; that no money was paid for such license except the regular fees paid by all applicants therefor; that no fraud, misrepresentation or mistake in any material regard was employed by any one or occurred in order that such should be conferred. Every license, or if lost, a copy thereof, legally certified so as to be admissible as evidence, or a duly attested transcript of the record of its conferment shall, before registering, be exhibited to the county clerk, who, only in case it was issued or indorsed as a license under seal by the regents shall indorse or stamp on it the date and his name, preceded by the words "Registered as authority to practice chiropody in the clerk's office of ....... county." The clerk shall thereupon give to every chiropodist so registered, a transcript of the entries in the register with a certificate, under seal, that he has filed the prescribed affidavit. The regents may, in their discretion and for cause deemed by them to be satisfactory, indorse as a license a certificate issued by the Pedic Society of the State of New York, prior to September 1, 1912, notwithstanding the failure of the holder thereof to cause the same to be registered prior to such date as required by the law then in force, provided application for such indorsement be made within three months after the taking effect of this act.

§ 279. **Persons not entitled to register unless holding a license.** No person shall be entitled to register as a chiropodist unless he or she shall hold the license provided for in section 272 of this article, or a certificate issued by the Pedic Society of the State of New York, and indorsed by the regents as provided in the preceding section. Every unrevoked certificate and indorsement of registry made as provided in this article, shall be presumptive evidence in all courts and places that the person named therein is legally registered. After September 1, 1912, no person shall register any authority to practice chiropody unless it has been issued or indorsed as a license by the regents. No such registration shall be valid unless the authority registered constituted at the time of the registration a license under the laws of the state then in force.

§ 2. This act shall take effect immediately.

Chap. 514, An Act to amend the public health law, in relation to the practice of pharmacy, as to working hours and sleeping apartments in pharmacies and drug stores.

§ 236. **Working hours and sleeping apartments.** No apprentice or employee in any pharmacy or drug store shall be required or permitted to work more than 70 hours a week. Nothing in this section prohibits working six hours overtime any week for the purpose of making a shorter succeeding week, provided, however, that the aggregate number of hours in any such two weeks shall not exceed 132 hours. The hours shall be so arranged that an employee shall be entitled to and shall receive at least one afternoon and evening off in each week and in addition thereto shall receive one full day off in two consecutive weeks. No proprietor of any pharmacy or drug store shall require any clerk to sleep in any room or apartment in or connected with such store that does not comply with the sanitary regulations of the local board of health. The provisions of this section alone regulate working hours and sleeping apartments in pharmacies or drug stores.

§ 2. This act shall take effect immediately.

